# Psychiatric Disorders and Alcohol Consumption Among Low-Income African Americans:Gender Differences

**DOI:** 10.3390/brainsci9040086

**Published:** 2019-04-18

**Authors:** Sharon Cobb, Shervin Assari

**Affiliations:** 1 School of Nursing, Charles R. Drew University of Medicine and Science, Los Angeles, CA 90059, USA; sharoncobb1@cdrewu.edu; 2 Department of Family Medicine, Charles R. Drew University of Medicine and Science, Los Angeles, CA 90059, USA

**Keywords:** race, gender, Blacks, African Americans, ethnic groups, psychiatric disorders, alcohol use

## Abstract

Background: Although cooccurrence of nonsubstance use disorders (non-SUDs) and substance use is well-established in the literature, most of what we know in this regard is derived from studies that have recruited predominantly White sample populations. As a result, there is a gap in knowledge on this link among low-income African Americans (AAs). There is also a need to understand how low-income AA men and women differ in these associations. Objective: To study whether there is an association between number of non-SUDs and amount of alcohol consumption by AA adults, and whether this association varies between AA men and women. Methods: This cross-sectional study recruited a nonrandom sample of 150 AA adults with non-SUDs (i.e., major depression, bipolar disorders, obsessive–compulsive disorder, paranoid disorder, panic disorder, posttraumatic stress disorder (PTSD), and schizoaffective disorder). The independent variable was the number of non-SUDs. The dependent variable was the amount of alcohol consumption. Age, socioeconomic status (educational attainment and household income), and self-rated health were covariates. Gender was the moderator. Linear regression models were used to analyze the data. Results: A higher number of non-SUDs was not associated with a higher amount of alcohol use in the pooled sample of AA adults. We, however, found a significant interaction between gender and number of non-SUDs on the amount of alcohol use, suggesting a stronger effect of non-SUDs on alcohol consumption in AA men than in AA women. Gender-stratified linear regression models showed a positive association between number of non-SUDs and amount of alcohol consumption in AA men but not in AA women. Conclusion: Non-SUDs impact alcohol use of AA men but not women. Future research should test whether AA men may have a higher tendency to turn to alcohol to regulate their emotions and cope with psychological pain due to multiple non-SUDs. The results also suggest that integration of services for SUDs and non-SUDs may be more relevant to provision of mental health services for AA men than AA women.

## 1. Background

Due to the cooccurrence between multiple psychiatric disorders [1], a large proportion of individuals have cooccurring disorders (COD) [2]. Dual diagnosis refers to CODs when a substance use disorder (SUD) and a non-SUD cooccur [3,4,5,6,7,8]. The cooccurrence between SUDs and non-SUDs is due to the common and shared risk factors. Overall, a wide range of social, behavioral [9], and biological [10] processes simultaneously increases risk of SUD and non-SUD conditions. For example, low socioeconomic status (SES) is a risk factor for anxiety disorders [11], major depressive disorder (MDD) [11], schizophrenia [12], and SUDs [13]. Another common risk factor is low SES neighborhoods, which is a proxy of environmental stressors and trauma [14] that concurrently increase risk of SUDs and non-SUDs [15]. Poor emotion regulation is also a nonspecific risk factor for SUDs and non-SUDs, as it has been linked to multiple psychiatric disorders [9], such as MDD [16], anxiety [17], and SUD [18]. Emotion dysregulation can be observed in various psychiatric problems [9]. However, most of what we know about CODs and dual diagnosis is derived from studies with predominantly White samples. As a result, less is known about comorbid SUDs and non-SUDs in African Americans (AAs) [19,20].

As culture and environment generate a context in which each racial and ethnic group lives, factors that shape development and manifestation of psychiatric disorders vary across groups. Thus, what applies to Whites may not necessarily be relevant to a historically marginalized group such as AAs. We should consider race and ethnicity as fundamental factors that change the patterns of co-occurring SUD and non-SUDs [2]. Using data from the Collaborative Psychiatric Epidemiology Studies (CPES), a study documented the AAs differ from Whites in the prevalence of cooccurring MDD, anxiety disorders, and SUDs. Prevalence of cooccurring psychiatric disorders was lower in AAs than Whites, among whom 5.4% and 8.2%, respectively, met diagnostic criteria for lifetime CODs. Similar to Whites, the majority of AA individuals with COD reported that symptoms of non-SUDs occurred before symptoms due to SUDs, suggesting that similarly in AAs and Whites, SUDs may be a coping response to non-SUDs. However, AAs differed from Whites in how COD was associated with unemployment among AAs but were less likely to have a history of psychiatric hospitalization [2].

In addition to the unique features that may distinguish AAs from Whites, considerable gender differences may exist in the associations between multiple non-SUDs and SUDs [21,22]. Even among White samples, not all studies have shown similar results [21,22]. In one study, attention deficit hyperactivity disorder (ADHD) was more strongly linked to SUD in men than women [21]. In another study on adolescents, while ADHD and conduct disorder were more likely to be comorbid with SUD in males than females, SUD and MDD showed a stronger association in males than females [22]. In a study, males and females did not differ in the association between SUDs and dysthymia and bipolar disorder [22]. Thus, even in studies among Whites, some but not all studies suggest that comorbidity between SUDs and non-SUDs differs between men and women. As a result, there is a need to study gender differences in dual diagnosis among AAs, particularly because the role of gender in shaping psychiatric disorders of AAs differs from that of Whites. The results may help clinicians who provide mental and substance use services and policy makers who need research-based results for the provision of mental health and substance use services for low-income AAs. Gender-specific patterns would advocate for considering gender in expanding mental health services for the low-income AA community, as some services would be more required in one than another gender.

Understanding the unique ways by which AAs experience comorbidity of multiple mental health problems is very important as these patterns have implications for how AAs seek help and utilize professional health care for their dual diagnosis [2]. However, as said before, most of the existing knowledge on cooccurrence of SUDs and non-SUDs of what we know on these processes are for the majority group (Whites). As gender differently correlates with social and mental health of AAs compared to Whites, there is also a need to compare the cooccurring SUDs and non-SUDs in AA men and AA women.

## 2. Aims

This study aimed to investigate the association between the number of non-SUDs and the amount of alcohol consumption by AA adults, and whether this association varies between AA men and AA women. Regarding aim 1, we hypothesized a positive association between non-SUDs and the amount of alcohol consumption by AA adults. Regarding aim 2, we expected a stronger positive association between the number of non-SUDs and the amount of alcohol consumption by AA men compared to women.

## 3. Methods

### 3.1. Design and Setting

A cross sectional study was undertaken to assess AAs with serious mental illnesses who were recruited from South Los Angeles using nonrandom sampling. The setting for this study was a mid-size community clinic in the South Los Angeles area, which has a high density of AAs. The surrounding environment of the clinic has been characterized with high rates of unemployment and poverty, substance abuse, and violence. The clinic provides primary health care services for low-income and uninsured individuals, specifically focusing on those with incomes below 200% of the federal poverty line.

### 3.2. Ethics

The study was approved by the University Human Subjects Protection Committee at University of California, Los Angeles. Participants provided verbal consent. Participants who completed the survey received a $15 gift card compensation.

### 3.3. Participants and Sampling

One hundred and fifty community dwelling aging adults were enrolled in this study. Individuals were eligible for participation in the study if they self-identified as Black or African American, at least 45 years of age or older, speak English, and diagnosed with a serious mental illness for at least one year. Participants were excluded if they were diagnosed with clinical dementia or Alzheimer’s disease.

### 3.4. Recruitment

Recruitment strategies included culturally-sensitive posters and flyers in the entrance of the mental health clinic. Mental health social workers employed at the clinic also referred participants for the study if they met the inclusion criteria.

### 3.5. Data Collection

Data collection was conducted over a period of 6 months. Data collection was conducted in a private space. In-person interviews were used to collect data, with special consideration for those with lower literacy skills and vision impairment. All study materials, such as the survey questionnaire, were developed at the fifth-grade reading level.

### 3.6. Measures

#### 3.6.1. Demographic Factors

Age and gender were the study covariates. Age was treated as an interval variable ranging from 45 to 78. Gender was treated as a dichotomous measure (male 1 female 0).

#### 3.6.2. Educational Attainment

Participants’ educational attainment was recorded as a nine-level categorical variable: (0) Below 8th Grade, (1) 9th Grade, (2) 10th Grade, (3) 11th Grade, (4) 12th Grade, (5) Associate Degree, (6) Bachelor’s Degree, (7) Master Degree, and (8) Ph.D./M.D./J.D. Degree. 

#### 3.6.3. Household Monthly Income

Participants were asked “Which of the following describes your combined monthly household income?” They were asked to include all employment wages, income from Social Security Disability Insurance, and other resources. Responses were on the following seven categories: (0) $0–500/month, (1) $600–900/month, (2) $1000–1400/month, (3) $1500–1900/month, (4) $2000–2400/month, (5) $2500–2900/month, and (6) $3000+/month.

#### 3.6.4. Number of Psychiatric Disorders

Participants were asked about the presence of (1) MDD, (2) bipolar disorders, (3) paranoid disorder, (4) posttraumatic stress disorder (PTSD), (5) obsessive–compulsive disorder (OCD), (6) panic disorder, and (7) schizoaffective disorder. The responses were either yes or no. We calculated a total psychiatric comorbidity score with a potential range between 0 to 7, with a higher score indicating multiple comorbid psychiatric conditions.

#### 3.6.5. Self-Rated Health (SRH)

Participants were asked to report their overall health as excellent, good, fair, or poor [23]. We treated SRH as an ordinal variable with responses that potentially ranged from 1 to 5, with a higher score reflecting poorer SRH. Poor SRH predicts all-cause mortality in the general [24,25] and clinical [26] population. In addition, reviews have shown that these effects are robust and SRH is a valid health measure, as it predicts mortality above and beyond confounders such as SES and health [23].

#### 3.6.6. Amount of Alcohol Consumption

The study participants were questioned with three items regarding their alcohol use. The first item asked if they drink alcohol. The second item asked how many days in the past month they consumed alcohol. The third item asked them how many drinks they consume on average per occasion. Then, monthly alcohol consumption was calculated by multiplying frequency and quantity [27,28,29,30,31]. Previous studies have used a similar approach to calculate the amount of alcohol use and have shown high reliability in self-reports [27,28,29,30,31]. Correlation coefficient between the two items was 0.443.

### 3.7. Statistical Analysis

We applied the software SPSS 23.0 for Windows (IBM Inc., Armonk, NY, USA) for data analysis. For description of the overall sample as well as by gender, we reported mean and standard deviation (SD). To explore bivariate correlations, we calculated zero order correlations using the Pearson test in the pooled sample and by gender. We also used an independent samples *t*-test to compare our descriptive characteristics between AA men and AA women. Finally, we applied linear regression models to conduct our multivariable analyses. First, we tested the collinearity between our predictors. Then, we tested the linear distribution of the error terms for our model. We ran four linear regression models, numbered consecutively from Model 1 to Model 4. The first two models (Model 1 and Model 2) were conducted in the overall sample that included both AA men and AA women. The last two models (Model 3 and Model 4) were conducted specific to each gender. The difference between the first and second model was that the first model only included the main effects; however, the second model also included the interaction between gender and number of non-SUDs. In all models, number of non-SUDs was the independent variable, amount of alcohol use was the dependent variable, age, education, household income, and SRH were the covariates, and gender was the effect modifier (moderating variable). From our regressions, we reported b, B, SE, and *p* values.

## 4. Results

### 4.1. Descriptive Statistics

This study included a total sample of 150 AAs with serious non-SUD(s). This number was composed of 49 AA men and 101 AA women.

Table 1 shows the results of descriptive information in the pooled sample as well as groups by gender. This table also compares AA men and AA women for demographic, SES, health status, and alcohol use. As this table shows, AA women were older than AA men in the study. AA men reported a higher amount of alcohol consumption than AA women. AA men and women did not differ in SES, SRH, and number of non-SUDs.

### 4.2. Bivariate Correlations

Table 2 shows the correlation between all the study variables in the pooled sample as well as for AA men and AA women. As this table shows, the number of non-SUDs was correlated with the amount of alcohol use in AA men but not in the pooled sample or AA women.

**Figure 1:**
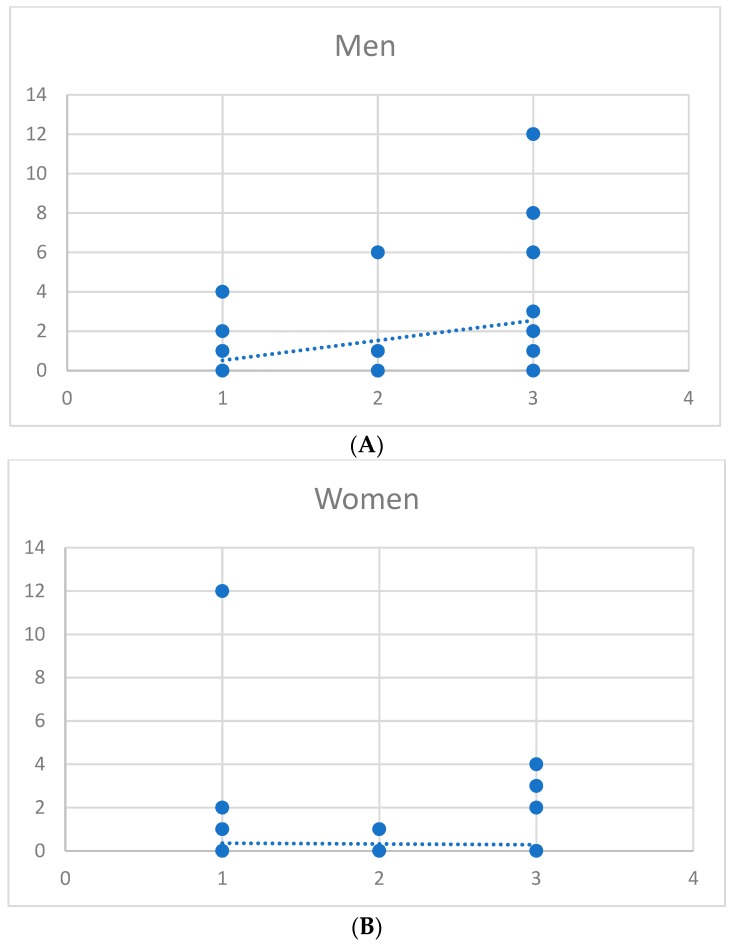
Correlation between number of psychiatric disorders and amount of alcohol drinking in African American men (**A**) and African American women (**B**). The x axis shows the number of psychiatric disorders and the y axis is the amount of alcohol use.

Figure 1 shows the correlation between the number of psychiatric disorders and the amount of alcohol drinking in African American men (Figure 1A) and African American women (Figure 1B).

### 4.3. Linear Regressions in the Pooled Sample

Table 3 shows the results of two linear regression models with number of non-SUDs as the independent variable and amount of alcohol use as the dependent variable. Model 1 showed no association between number of non-SUDs and amount of alcohol consumption in the pooled sample. Model 2, however, showed a significant interaction between gender and number of non-SUDs on the amount of alcohol use, suggesting a stronger association in AA men than in AA women.

### 4.4. Linear Regressions by Gender

Table 3 also summarizes two gender-stratified models specifically for AA women (Model 3) and AA men (Model 4). These models suggest an association between the number of non-SUDs and the amount of alcohol use in AA men but not in AA women.

## 5. Discussion

The current study explored the association between the number of non-SUDs and the amount of alcohol use in a community sample of low-income AA adults with serious mental disorder(s). While low-income AA men with multiple non-SUD mental disorders consumed more alcohol, this association could not be found among low-income AA women with non-SUD psychiatric disorders.

This is not the first study to explore the association between non-SUDs and alcohol use. The result (no association in the pooled sample) was unexpected given the well-established literature on dual diagnosis and the link between non-SUDs and SUDs and is not in line with previous research which has mostly focused on Whites [3,4,5,6,7,8]. As our association was only significant in AA men, our negative finding in the pooled sample can be attributed to the fact that we had more AA women than AA men in this study. Still, our findings of not supporting the literature focused on predominantly Whites re-emphasizes the need for considering group differences in risk factors, manifestations, and consequences of psychiatric disorders. Instead, this study’s results reveal literature in this area may apply to middle-class Whites but not necessarily among low-income AAs. Research should also recruit larger samples of AAs and members of other racial and ethnic groups into studies focusing on psychiatric disorders. These differences may be in part attributed to the role of race and ethnicity as a social context, in which causes and consequences of psychiatric disorders emerge [2,3]. These differences suggest that disregarding race and ethnicity in both research and clinical practice may be associated with misdiagnosis, mistreatment, stigma, and poor quality of care for AA community, leading to health disparities [32,33]. It is also significant to note the historical and social contexts of the AA population, who are heavily affected by social adversities, racism, poverty, and lack of access and poor physical health [34].

Patterns of comorbidities between SES, physical health problems, health behaviors, and psychiatric disorders in AAs differ from those of Whites [35,36,37]. For example, obesity is a weaker risk factor for MDD for AAs than for Whites [38,39,40,41]. SES indicators in general, and educational attainment in particular, have smaller protective effects on MDD in AAs and Whites, with highly educated AAs being at higher risk of MDD [42]. In a longitudinal study, highly educated AA men but not AA women or White men or women were at high risk of future depressive symptoms [43], a finding which was replicated for AA youth over time [44]. Another study revealed a higher risk of MDD in high-income AA men but not AA women [45]. These findings were also replicated [46,47]. In another study, education and income improved the mental health of Whites but not AAs [48]. Furthermore, educational attainment reduced the risk of psychological distress and depressive symptoms for AA women but not men [49].

Previous studies have documented race and race by gender variation in the correlations between MDD, anxiety, alcohol use, substance use, suicidality, and perceived health, perceived mental health need, and service use [35,37,43,50,51]. The Fragile Families and Child Well-Being compared AA (*n* = 2407) and White (*n* = 894) individuals. Self-rated health (SRH) was consequently measured four times at baseline, year 1, year 3, and year 5. AAs and Whites were similar regarding the effects of GAD and SUDs (alcohol usage issues) on SRH at baseline and over the 5 years of follow-up. The study, however, documented cross-racial differences in the combined (additive) effects of GAD and MDD. For AAs, MDD and GAD were associated with a worse trajectory of SRH over time. For non-Hispanic Whites, while MDD predicted worse baseline SRH, anxiety was associated with better SRH at baseline and over time. Among non-Hispanic Whites, anxiety predicted a worse trajectory of SRH; however, MDD did not correlate with SRH [50]. In another cross-sectional study that used data from the National Survey of American Life 2003, which included a nationally representative household probability sample of AAs (*n* = 3570) and Caribbean Blacks (*n* = 1621), for AAs, anxiety and MDD had independent (i.e., separate) effects on perceived mental health. For Caribbean Blacks, however, MDD but not anxiety independently affected perceived mental health. When the joint (e.g., combined) effects of anxiety disorder and MDD were explored, GAD but not MDD impacted poor mental self-rated health [37]. Additionally, in another study with a sample size of 6147 suicide attempts (5388 Whites and 759 AAs), AAs and White individuals who were hospitalized for suicide attempt did not differ in gender and age; however, they differed by insurance type. While AAs were more likely to have federal insurance (Medicare and Medicaid), White individuals were more likely to have private health insurance. AAs who were admitted to hospital for a suicidal attempt were more likely than their White counterparts to have obesity, while White individuals who were hospitalized with a suicide attempt were more likely than AAs with suicide to be underweight. Although the frequency of psychiatric disorders (i.e., MDD, SUDs, and psychotic disorders) was equally distributed between AAs and Whites who were hospitalized for a suicide attempt, AAs had more comorbid medical conditions than their White counterparts. Compared to Whites, AAs were more likely to have chronic diseases such as obesity, chronic renal disease, hypertension, chronic obstructive pulmonary disease, hematological conditions (i.e., coagulopathy), and obesity, while compared to AAs, Whites were more likely to have other neurological disorders [35].

This study is not the first to show that men may have a higher tendency to show an association between non-SUDs and use of substances (i.e., alcohol) [21,22]. This study, however, is one of the first to establish similar gender differences in the association between number of non-SUDs and amount of alcohol use by low-income AA men with a serious psychiatric disorder. Our results extend the literature on dual diagnosis and multimorbidity of SUD and non-SUDs to low-income AA men. This information is useful to clinicians who serve this population as limited knowledge exists regarding mental health needs of AA men with multiple psychiatric disorders [2,3].

Previous research has suggested that AA men may be at an increased risk of SUDs and non-SUDs. In a study, Compton et al. (2006) examined racial variation in the changes in the prevalence of MDD and comorbid SUD, comparing data from two cross-sectional national surveys with representative samples of American adults: National Longitudinal Alcohol Epidemiologic Survey (NLAES; 1992) and National Epidemiologic Survey on Alcohol and Related Conditions (NESARC; 2002). They found an increase from 1992 to 2002 in the prevalence of MDD from 3% to 7%, a change which was statistically significant for non-Hispanic Whites, Hispanics, and AAs. In fact, AA men were the only group who experienced an increase in rate of comorbid MDD and SUD over time [20].

Some evidence suggests that MDD and some other psychiatric disorders are qualitatively different for AA men than women [52,53]. In a study by Watkins and colleagues, AA women reported AA men’s depression to be qualitatively different. They mentioned that depression is a phenomenon specific to AA men’s culture and gender. Such gender- and culture-specific approaches may be superior to strategies that ignore gender-specific culture of the target group. In addition, depressive symptoms and distress that may not necessarily meet the Diagnostic and Statistical Manual of Mental Disorders (DSM) criteria require investigation [52,53].

The finding of this study may be understood with regard to gender socialization. Regardless of race, overall, men may tend to externalize their symptoms (i.e., substance use), while women have a tendency toward internalizing disorders (i.e., depression, anxiety). These gender differences may be due to socialization processes (including the attitude of the society regarding traditional gender roles and behaviors) and have been shown across cultures. These patterns shape gender differences in emotion regulation, help seeking, and other coping behaviors [54]. As a result of these differences, males and females learn to respond differently to stress and distress, differences that have clinical implications and should not be overlooked by clinicians. If such variations are ignored, clinicians may expect similar presentation of psychiatric disorders in men and women. Such expectations may result in a systemic underestimation of psychiatric disorders such as depression and anxiety in men, particularly because men have a lower tendency to disclose their emotional symptoms, and delay their help seeking. In other words, the presence of behaviors such as alcohol use should not obscure men’s non-SUDs, as mood disorders, anxiety disorder, and other psychiatric disorders may accompany SUDs in men [55].

Due to historical racism, marginalization, legacy of slavery, and other related social and historical processes, the AA community has lower levels of trust and acceptability toward participation in psychiatry research [56,57,58]. AAs’ trust toward the US health care system may still be wary due to unethical exploitation of the AA community, exhibited in the Tuskegee experiments [59] and the case of Henrietta Lacks and the HeLa cells [60]. Another major reason is lower quality and access of mental health treatment for AAs than Whites. For example, historically, AA [61,62,63] and low-SES [64] patients with bipolar disorder and other psychiatric disorders [65] have been misdiagnosed and managed inappropriately. This may be attributed to the mental health movement in the 1960s, when AA men were more likely to be diagnosed with schizophrenia if they exhibited activism for civil rights [66]. This led to the criminalization of mental health in the AA community, which may be an attributing factor for underutilization of adequate treatment for mental illness and symptoms.

As a result of systemic under-representation of AA in psychiatry research, there is a systemic lack of information on how to diagnose and treat psychiatric conditions in the AA community [67]. This is just one of the many reasons that mental health outcomes of AAs with psychiatric disorders are worse [51]. As a result, the burden of identical psychiatric disorders is higher for AAs than for Whites [51]. That is, psychiatric disorders are more disabling and chronic in the AA community compared to Whites. For example, MDD is associated with a stronger hit on economic status [36] and self-esteem [68] of AAs than Whites. As a result, screening, diagnosis, and treatment of psychiatric disorders such as MDD in AA may require attention to other social and environmental contexts in which AAs with the disorder live [36].

AAs with mental health needs, particularly AA men with psychiatric disorders, are still experiencing discrimination across institutions including but not limited to the health care system. Systemic structures have to be altered to recognize and confront racism as both an internal and external factor affecting the US health care system. Highly educated AAs experience greater rather than reduced discrimination. They are also likely to bear the responsibility for the social and economic survival of their families, including distant relatives and kinship networks, due to higher incomes and education levels, leading to deterioration of their mental health [69]. For these reasons, high-SES AAs may minimize their symptoms, resulting in considerable unmet needs. In addition, they may possess higher levels of fear of discrimination based on race and mental illness. Individuals with psychiatric disorders may fear being perceived as “weak” [70]. Even though this sample reports lower income levels, they may still experience the same effects as higher-SES AAs, which will be heightened by poverty. Racism has caused mistrust in the Black community regarding the healthcare system, particularly among those who require mental health treatment. Patients and community members believe that antiracism education and community-driven practice are important elements if the system wishes to reduce unmet need of AAs with mental problems. In addition to the society as a whole, it is important for mental health systems to confront the racism that exists historically and contemporarily [71] and implement culturally competent strategies.

Another issue is that mental health needs of the AA community are neglected due to the lack of provision of culturally sensitive mental health services to economically disadvantaged urban areas. Urban living seems to tend to increase the biological or social/environmental risk of multiple psychiatric disorders, such as psychosis, GAD, and MDD. It is important to note that our study was conducted in an urban neighborhood that has been impoverished and under-resourced for decades. Therefore, this study interestingly represents the factors that are existing among this vulnerable population. Despite the growing mental health needs of urban populations, access to mental health services is still a challenge [72,73,74], particularly for individuals who are struggling with poverty.

These are all consequential processes, as mistrust is a historical barrier against care seeking of AAs with psychiatric conditions, impacts the quality of their relationship with physicians, and ultimately leads to nonadherence [72,73].

### 5.1. Clinical and Public Health Implications

The results reported here have many implications for research, practice, and policy. Integration of treatment of substance use and other psychiatric disorders may be more necessary for AA men than AA women [74]. Particularly for men, SUDs are highly comorbid with MDD, anxiety, psychotic disorder, antisocial personality disorder, and borderline personality disorder. Comorbidity of SUDs and other psychiatric disorders worsens the outcome and prognosis of treatment of such disorders [74,75,76]. Research investigators should replicate these findings using larger sample sizes with nationally representative samples. Further research may also explore factors that explain gender differences observed in this study. One hypothesis is that men generally have a higher tendency to use alcohol and substances to cope with stress, including psychological pain due to non-SUDs.

### 5.2. Limitations

This study had a few limitations. First, due to a cross-sectional design, causal inferences are not plausible. Second, the sample was a convenient sample. As a result, generalizability of the results is limited. Third, the sample size was very small. The sample size was also not equal between men and women. The current study used a self-reported measure of psychiatric disorders. However, as we sampled our participants from individuals with a psychiatric disorder (PD), their self-reported psychiatric disorders are likely to be valid. Future research should replicate these findings using larger sample size, random sampling, and using administrative data, or structural interviews. The results should be considered as preliminary and interpreted with caution.

## 6. Conclusions

In summary, the number of non-SUDs is associated with the amount of alcohol consumption in low-income AA men but not low-income AA women. Future research should test whether AA men have a higher tendency to use alcohol to regulate their emotions and cope with their psychiatric disorders. The results may also suggest that integration of services for SUDs and non-SUDs may be more relevant to mental health provision of services to low-income AA men than low-income AA women with multiple psychiatric disorders.

## Figures and Tables

**Table 1:** Correlation between all the study variables in the pooled sample as well as African American men and women.

	All *N* = 150		African American Women *n* = 101		African American Men *n* = 49	
	Mean	SD	Mean	SD	Mean	SD
Age *	55.78	7.40	54.87	7.40	57.65	7.10
Educational Attainment	3.61	1.40	3.53	1.51	3.76	1.13
Household Income	1.37	1.50	1.39	1.41	1.35	1.67
Self-Rated Health (Poor)	3.61	1.01	3.59	1.03	3.65	0.97
Number of Psychiatric Disorders	2.07	0.89	2.09	0.88	2.02	0.90
Amount Alcohol Use *	0.72	2.11	0.32	1.38	1.55	2.98

SD; Standard deviation; * *p* < 0.05 (Independent sample *t* test).

**Table 2:** Bivariate correlation matrix in the pooled sample and by gender.

	1	2	3	4	5	6	7
**All (*N* = 105)**							
1 Gender (Male)	1.00	0.18 *	0.07	−0.01	0.03	−0.04	0.28 **
2 Age		1.00	0.08	0.05	0.11	−0.11	0.02
3 Educational Attainment			1.00	0.17 *	−0.07	−0.11	−0.07
4 Household Income				1.00	−0.23 **	−0.24 **	−0.02
5 Self-Rated Health (poor)					1.00	0.20 *	0.01
6 Number of Psychiatric Disorders						1.00	0.12
7 Amount Alcohol Use							1.00
**African American Men (*n* = 49)**							
2 Age		1.00	0.02	−0.05	0.07	−0.20 *	0.01
3 Educational Attainment			1.00	0.13	−0.06	−0.07	−0.04
4 Household Income				1.00	−0.30 **	−0.31 **	−0.10
5 Self-Rated Health (Poor)					1.00	0.36 **	0.06
6 Number of Psychiatric Disorders						1.00	−0.02
7 Amount Alcohol Use							1.00
**African American Women (*n* = 101)**							
2 Age		1.00	0.21	0.25	0.18	0.10	−0.08
3 Educational Attainment			1.00	0.28	−0.12	−0.22	−0.18
4 Household Income				1.00	−0.10	−0.12	0.04
5 Self-Rated Health (Poor)					1.00	−0.13	−0.06
6 Number of Psychiatric Disorders						1.00	0.31 *
7 Amount Alcohol Use							1.00

* *p* < 0.05; ** *p* < 0.01.

**Table 3:** Summary of linear regressions in the pooled sample.

	Unstandardized B	Std. Error	Standardized Beta	*t*	*p*
**Model 1 (All; *N* = 150)**					
Gender (Male)	1.29	0.36	0.29	3.56	0.00
Age	0.00	0.02	−0.01	−0.12	0.91
Educational Attainment	−0.12	0.12	−0.08	−0.97	0.34
Monthly Household Income	0.02	0.12	0.02	0.21	0.84
Self-Rated Health	−0.06	0.18	−0.03	−0.35	0.73
Number of Psychiatric Disorders	0.31	0.20	0.13	1.58	0.12
Constant	0.42	1.51		0.27	0.78
**Model 2 (All; *N* = 150)**					
Gender (Male)	−0.93	0.91	−0.21	−1.03	0.31
Age	−0.01	0.02	−0.05	−0.61	0.54
Educational Attainment	−0.09	0.12	−0.06	−0.77	0.44
Monthly Household Income	0.01	0.12	0.01	0.11	0.91
Self-Rated Health	0.06	0.18	0.03	0.33	0.74
Number of Psychiatric Disorders	−0.09	0.25	−0.04	−0.36	0.72
Number of Psychiatric Disorders * Gender	1.10	0.41	0.56	2.66	0.01
Constant	1.38	1.53		0.91	0.37
**Model 1** **(African American Women; *n* = 101)**					
Age	0.00	0.02	−0.02	−0.18	0.858
Educational Attainment	−0.03	0.09	−0.03	−0.31	0.758
Monthly Household Income	−0.11	0.11	−0.11	−1.00	0.318
Self-Rated Health	0.07	0.15	0.05	0.46	0.649
Number of Psychiatric Disorders	−0.13	0.18	−0.08	−0.71	0.481
Constant	0.78	1.32		0.59	0.556
**Model 1** **(African American Men; *n* = 49)**					
Age	−0.05	0.07	−0.12	−0.75	0.457
Educational Attainment	−0.35	0.41	−0.13	−0.84	0.406
Monthly Household Income	0.26	0.27	0.15	0.95	0.345
Self-Rated Health	0.00	0.46	0.00	0.00	0.998
Number of Psychiatric Disorders	1.01	0.50	0.31	2.02	0.050
Constant	3.29	3.88		0.85	0.401
